# Monitoring and assessment of potentially hazardous particles in high-risk workplaces in the production of Ni-Cd batteries

**DOI:** 10.1038/s41598-025-33967-8

**Published:** 2026-01-06

**Authors:** Michaela Tokarčíková, Šárka Bernatíková, Pavel Rössner, Kristyna Vrbova, Michal Šíma, Roman Gabor, Petr Běčák, Jana Seidlerová

**Affiliations:** 1 Nanotechnology Centre, Centre for Energy and Environmental Technologies, VSB-Technical University of Ostrava, 17. listopadu 15/2172, 708 00 Ostrava, Poruba, Czech Republic; 2 Department of Fire Protection, Faculty of Safety Engineering, VSB-Technical University of Ostrava, 708 00 Ostrava, Czech Republic; 3 Department of Toxicology and Molecular Epidemiology, Institute of Experimental Medicine CAS, Prague, Czech Republic; 4 Faculty of Material Science and Technology, VSB-Technical University of Ostrava, 17. listopadu 15/2172, 708 00 Ostrava, Poruba, Czech Republic

**Keywords:** Ni-Cd battery, Battery production, Respirable particles, Monitoring, Toxicity, Chemistry, Environmental sciences, Health occupations, Risk factors

## Abstract

The manufacture of Ni-Cd batteries involves the risk of contact with and inhalation of dust particles containing heavy metals, including potential carcinogens. Three workplaces were selected that appeared to pose the greatest risk to workers in terms of exposure to heavy metal (nickel and cadmium) dust generated during the production and handling of nickel-cadmium plates. Although the limits for dust and carcinogens and mutagens are very strict, they are gradually being tightened. Therefore, the main motivation was to find the source of the highest amounts of respirable particles, determine the size of the particles, the composition of the fraction and evaluated potential toxicity of particles captured on the filters. This will help to propose additional measures to minimise concentrations of potentially carcinogenic particles in the working environment. The chemical analysis, particle size distribution, metal content and cytotoxicity of the particles trapped on the filters were investigated. While the concentration of heavy metals was well below the permissible exposure limits, the extracts obtained from the sampled filters had a significant effect on cytotoxicity, particularly those containing lower concentrations of particles.

##  Introduction

Production in the industrial sector, including the manufacture of fertilisers, paper, pulp, textiles, tanneries and batteries production and others is still increasing. This has a direct or indirect negative impact on the environment or human health due to the release of harmful metals during the production process^1^. In view of the requirement to reduce greenhouse gas emissions and reduce the amount of fossil fuel combustion, there is a growing requirement to use electricity generated from renewable sources. Especially, the battery production sector is experiencing rapid growth. Although Ni-Cd batteries are gradually being replaced by Ni-MH and Li-ion systems, they are still being produced. Ni-Cd batteries are used as an alternative energy source in transport applications due to their long shelf life and ability to operate stably in a temperature range of -20 to + 50 °C (with extremes of -50 °C to + 70 °C for short periods). The typical composition of Ni-Cd batteries is 29–40 wt% Fe, 15–20 wt% Ni and Cd, and trace amounts of Al, Co, Cr, Mn and Zn^2^. The phase composition, construction and the dimensions of the Ni-Cd batteries depend on their intended application.

In general, metals are among the oldest toxins to have a negative impact on the environment and human health^3^. The most common heavy metal contaminants in the environment are arsenic, cadmium, lead, mercury, chromium, nickel and copper^4^. While some metals are known to cause cancer and organ damage, while the toxic effects of others have not yet been clearly assessed^5^. In general, the reactive oxygen species (ROS) are considered to be the most important factor in the carcinogenicity of metals. At normal physiological concentrations, ROS play a key role in activating certain intercellular and intracellular signalling pathways that are involved in processes such as proliferation, apoptosis, autophagy and other biological processes. On the other hand, ROS are toxic to the cells because they contribute to high levels of oxidative stress in the organism. This can damage biomolecules including DNA, leading to cell dysfunction or death, and causing an imbalance between oxidants and antioxidants^3,5^.

Ni a Ni-containing compounds are naturally distributed in the Earth’s crust. They are released into the atmosphere through various natural processes. The emission of Ni is increasing due to its high industrial use, leading to unavoidable environmental pollution. Ni is recognised as an essential element for biological processes, such as the healthy growth of plants and animals, as well as for soil and microbial life^6^ and is also present in small amounts in human tissue^7^. However, its essentiality in human health is still in doubt, instead, its exposure can cause severe and acute adverse health effects. The toxicological behaviour of Ni compounds depends on their solubility and releasing of Ni^2+^ ions^8^, bioavailability^9^, physicochemical properties such as size, distribution, agglomeration and surface properties of particles^10^, and the amount, duration and the route of exposure^11^. The main routes of Ni exposure are inhalation, ingestion and dermal contact^6^. Chronic exposure to nickel dust or vapour in humans most commonly occurs through welding nickel alloys, contributing to all type of respiratory disorders, higher incidences of pulmonary and nasal cancers, as well as reproductive health issues^12^. Although it is difficult to determine the specifications of Ni compounds, regular exposure to Ni affects multiple organs of living systems in people working in industries such as refining, mining and metallurgy, the stainless-steel industry and battery manufacturing facilities^12^.

Cadmium occurs naturally in a small amount as an impurity in zinc and lead ores. The most common Cd application is in the manufacture of Ni-Cd batteries, in the glass industry, as an anti-corrosion agent, as preservative in metal allow production and refinement of zinc, copper and lead ores^5^. Cadmium is a potential carcinogen and due to its strong toxicity, high mobility and persistence, poses a threat to the environment and human health. Exposure to Cd accumulates in the liver and kidneys and has a negative effect on various organ systems in the human body^13^. Furthermore, Cd is one of the most water-soluble elements, and its degradation by microorganisms is very difficult. The half-time of its elimination from the human body is approximately 20–40 years^5^.

Cobalt is a relatively widespread element on Earth commonly used in metallurgy and the glass industry as well as in catalysts. In small amounts, cobalt is beneficial to the human body because it is part of vitamin B12, which is responsible for the proper production of red blood cells^14^. On the other hand, other cobalt compounds have been described as toxic to the environment and the human body after excessive exposure^15^. The Co^2+^ form is responsible for cobalt toxicity. Co toxicity is difficult to detect and is therefore often underdiagnoses^16^. Due to its widespread occurrence, people can be exposed to Co compounds in everyday life through inhaling ambient air, consuming and drinking water containing Co, or through occupational exposure in manufacturing processes^15^.

Iron is the fourth most abundant element in the Earth’s crust and is the most widely used base metal in everything from consumer goods to electronics to steel structures. Iron is also an essential trace element that helps to metabolise proteins, supports cell health and is involved in many enzymatic reactions and, most importantly, in the production of haemoglobin^17^. Iron has the ability to generate reactive oxygen species, which can damage cellular macromolecules. This makes iron a potential hazard in terms of toxicity^18^. Conversely, a study by^19^ shows that the presence of Fe may reduce the Cd body burden in the organisms.

It is not only potentially carcinogenic particles that pose a risk to human health and the environment, but also particulate matter (PM) pollution. The main sources of PM pollution in enclosed production halls are for example grinding, fine cutting and welding operations. The negative impact of dust on the human body depends on the origin of the dust, its properties, particle size, air concentration, exposure time and conditions. PM is categorised by particle size into coarse (< 10 μm diameter), fine (< 2.5 μm) and ultrafine (< 1 μm). Coarse particles (PM10) which derive from natural and industrial sources are trapped in the upper respiratory tract and generally do not penetrate beyond the upper bronchus. Fine (PM2.5) and ultrafine (PM1) particles which are produced by the combustion of fossil fuels, penetrate the lower respiratory tract, and are therefore, more dangerous than PM10 particles. Especially, particles lower than 1 μm (PM1) can easily reach the alveoli and particles smaller than 0.5 μm can penetrate the bloodstream and affect individual organs directly^20^.

The toxicity and negative impact of particles depend on several factors, such as particle size distribution, surface area, agglomeration state, shape, charge and surface chemistry and the loading amount. The simultaneous effect of these factors must also be considered^21,22^.

The manufacture of Ni-Cd batteries is associated with the release of dust particles containing heavy metals, including potential carcinogens. Directive 2004/37/EC^23^ of the European Parliament and of the Council deals with the protection of the health and safety of workers against risks arising from exposure to carcinogens, mutagens or substances toxic to reproduction at work. Although the permissible exposure limit (PEL) for dust containing Ni and Cd is very strict, monitoring workplaces at higher risk is desirable. Monitoring can help to identify the main source of hazardous dust and eliminate the risk by implementing appropriate measures. The limit value for Cd and its inorganic compounds is 0.001 mg/m^3^ for the inhalable fraction over an 8-hour period. In addition, the limit values for nickel compounds have been tightened for Ni from 0.1 mg/m^3^ to 0.05 mg/m^3^ for the inhalable fraction and to 0.01 mg/m^3^ for the respirable (< 2 μm) fraction over an 8-hour period in January 2025. According to government regulations 41/2020 Sb^24^. , the PEL for cobalt and its compounds is 0.05 mg/m^3^ and the highest permissible concentration is 0.1 mg/m^3^ over 8 h. Although Fe is not classified as a dangerous metal, and Fe-dust is mostly non-specific in its effects, the limit value of respirable fraction of the aerosol is 10 mg/m^3^ over 8 h.

Three workplaces with the highest risk of particle exposure during Ni-Cd battery manufacturing were chosen for monitoring and sampling. Although dust is minimised, the working environment is clean and the limits are regularly checked with no deficiencies identified, the main reason for this monitoring is the ever-tightening limits for the maximum permissible concentrations of toxic and potentially hazardous metals in the working environment, with a tighter limit for Ni in 2025. Furthermore, workers are preventive gradually being transferred to other workplaces due to high levels of exposure to potentially hazardous dust. Sampling and analysing particles captured on filters during a work shift will provide a more accurate assessment of the risks associated with working with potentially hazardous materials. A comprehensive approach combining chemical analysis of captured particles with cytotoxicity testing allows for a more accurate assessment of the risks specific to a given workplace. Most importantly, it will identify locations with a higher concentration of potentially dangerous elements in the dust. This will enable measures to be taken in future to better protect employees´ health, not only by tightening maximum permitted limits. The paper evaluates the working environment in terms of monitoring maximum permissible concentrations and the composition of sampled dust. It also assesses the cytotoxicity of the collected samples. It does not aim to recommend corrective or preventive measures.

## Results and discussion

**Fig. 1:**
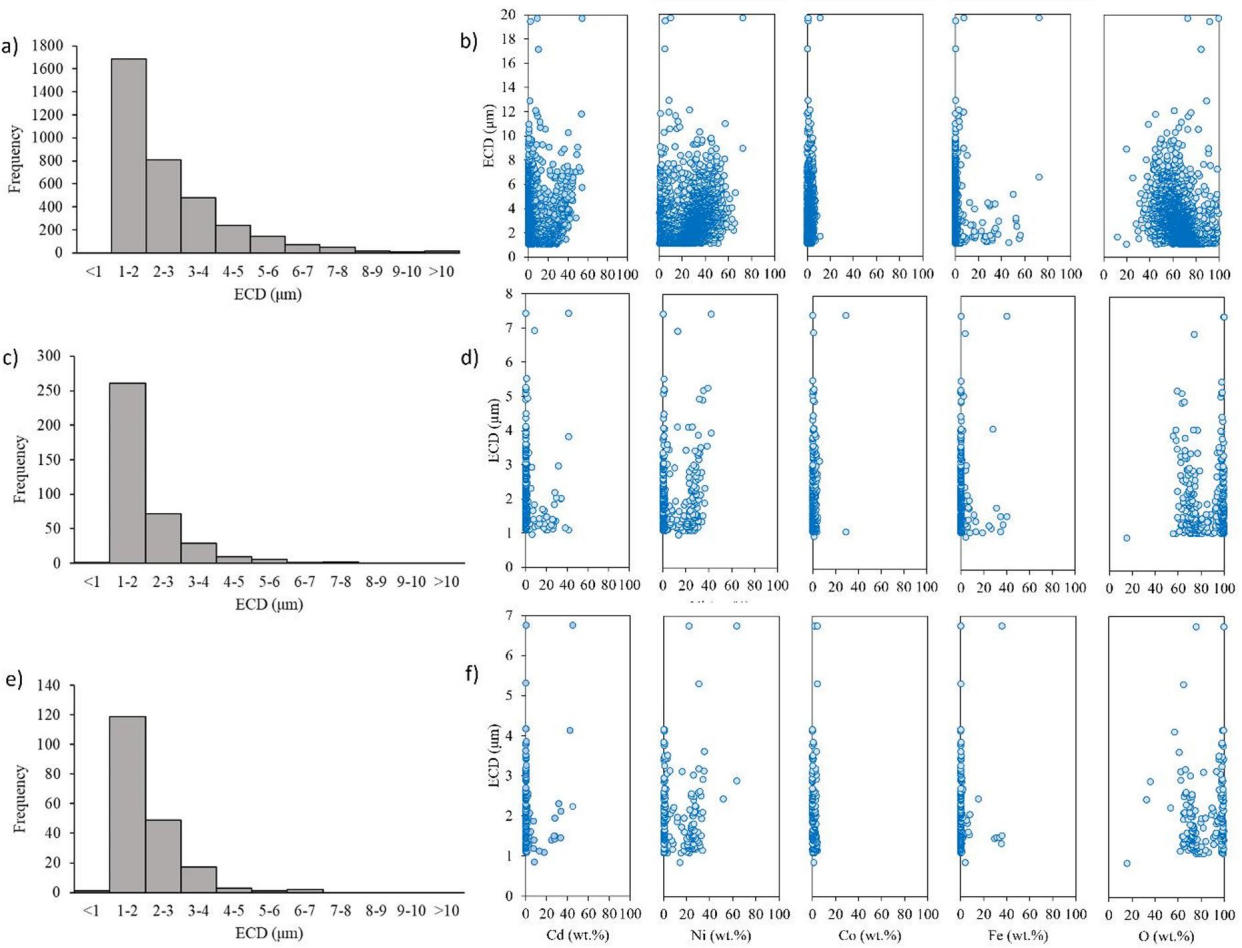
Frequency of particle size distribution on the filter sampling in the (**a**) Plastic line workplace, (**c**) APAM and (**e**) Briketka workplace. The weight% (wt%) of Cd, Ni, Co, Fe and O distributed across the different particles size ranges on the filter sampling in the (**b**) Plastic line workplace, (**d**) APAM and (**f**) Briketka workplace. ECD - Equivalent Circular Diameter (µm).

The highest number of particles was captured on the filter during the sampling in the Plastic line workplace equipped with one two-stage filter (see Table 1; Fig. 1a)). With increasing numbers of two-stages filters on the monitoring workplaces, the frequency of particles captured during sampling decreased significantly. The lowest particle concentration was determined on the filter sampled in the Briketka workplace, which is equipped with 9 of two-stage filters.

No ultrafine particles (PM1) were detected in any of the filters sampled in the monitored workplaces. However, the respirable fraction (< 2 μm) was the most abundant fraction on the filters: 47.8% for the filter sampled in the Plastic line workplace (Fig. 1a)), 68.8% for the APAM workplace (Fig. 1c)) and 62.5% for the Briketka workplace (Fig. 1e)). As particle size increased, their frequency and number on the filters decreased, due to their faster sedimentation and more efficient filtration of larger particles.

The diagram of the production hall including the workplaces where the sampling was carried out is described in more detail in the part Monitored workplaces.

Figure 1b), d) and e) shows the equivalent circular diameter (ECD) of Cd, Ni, Co, Fe and O determined using STEM on the filters sampled from the Plastic line (b), the APAM (d) and the Briketka (f) workplaces. Nickel was the most frequent element in all filter fractions, followed by cadmium, iron and cobalt. This finding was also confirmed by OES-ICP analysis of decomposed filters (Table 1).

**Fig. 2:**
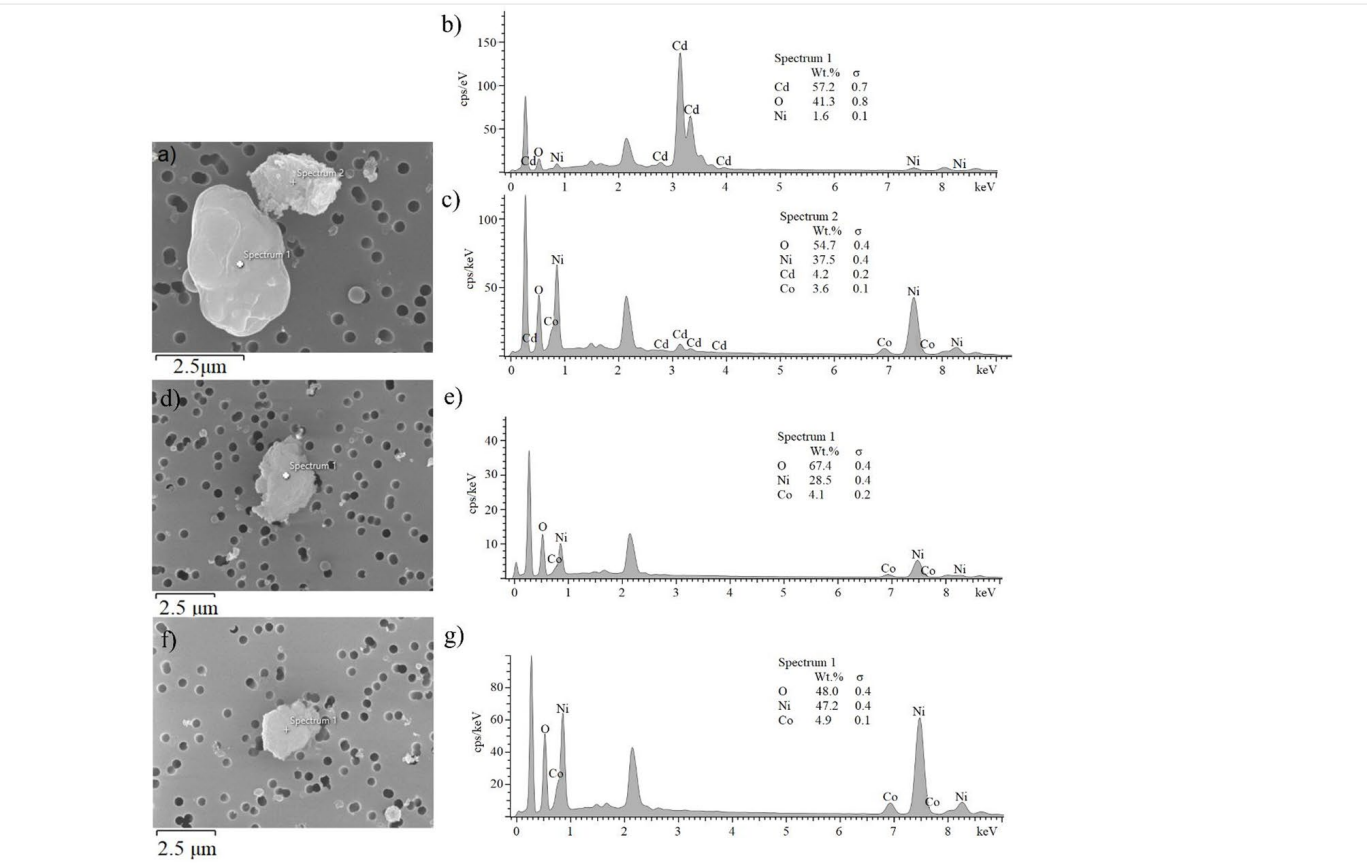
The SEM images of particles on the filter sampled in (**a**) Plastic line, (**d**) APAM and (**f**) Briketka workplaces. The SEM-EDS analysis of particles on the filter sampled in (**b**) and (**c**) Plastic line, (**e**) APAM, (**g**) Briketka workplaces.

Figure 2a)-g) shows the detailed SEM and EDS analysis of the particles present on the filters from the monitored workplaces. Figures 2d) and f) show the irregular shape and sharp edges of the nickel particles, whereas the cadmium particle has smoother, rounder edges (Fig. 2a), Spectrum 1).

Neither the EDX nor the OES-ICP analysis detected the presence of Cr, Zn, V, Mo, Al, Ba, Be, Mn and Pb in the filters. The concentrations of Ni, Co, Cd and Fe determined after the decomposition of the filters in acids (Table 1) are significantly lower than the permissible exposure limits established by the^24^ Directive Government Regulation No. 41/2020 which are 0.05 mg/m^3^ for Cd and Co, 0.5 mg/m^3^ for Ni and 10 mg/m^3^ for Fe.

**Table 1:** Concentrations of the monitoring elements determined in the extracts by OES-ICP (c_OES−ICP_ in µg/l) and the concentrations captured on the filters sampled in the plastic line, APAM and Briketka workplaces during the standard work shift (in mg/m^3^).

Monitored element	Workplace	Number of filters	c_OES−ICP_ (µg/l)	c_metal_ (mg/m^3^)
Ni	Plastic line	1	6000 ± 300	0.17
	APAM	5	512 ± 26	0.014
	Briketka	9	429 ± 21	0.012
Cd	Plastic line	1	576 ± 63	0.017
	APAM	5	42.3 ± 5	0.0012
	Briketka	9	33 ± 4	0.0009
Co	Plastic line	1	207.5 ± 10	0.006
	APAM	5	59 ± 3	0.0017
	Briketka	9	30 ± 2	0.0009
Fe	Plastic line	1	50 ± 3	0.0014
	APAM	5	100 ± 6	0.0029
	Briketka	9	20 ± 1	0.0006

The limits of detection (LOD) and quantification (LOQ) for OES-ICP are as follows: 0.541 µg/l and 6 µg/l for Ni; 0.199 µg/l and 2 µg/l for Cd; 0.096 µg/l and 1 µg/l for Co; 0.141 µg/l and 2 µg/l for Fe.

### Determination of cytotoxicity

Two commercially available tests were used to evaluate the potential negative biological impact of the compounds adsorbed onto the filters. The MTS assay monitors the conversion of the supplied reagent into formazan. The efficiency of this reaction allows to estimate the activity of cellular dehydrogenases which correlates with the cell viability. In contrast, the LDH test detects lactate dehydrogenase released into the culture medium. The activity of this extracellular enzyme is assessed by the rate of conversion of a provided substrate to formazan; the result is proportional to the extent of plasma membrane damage^25^. Therefore, decreased MTS and increased LDH levels will be detected for cells negatively impacted by the tested compounds.

**Fig. 3:**
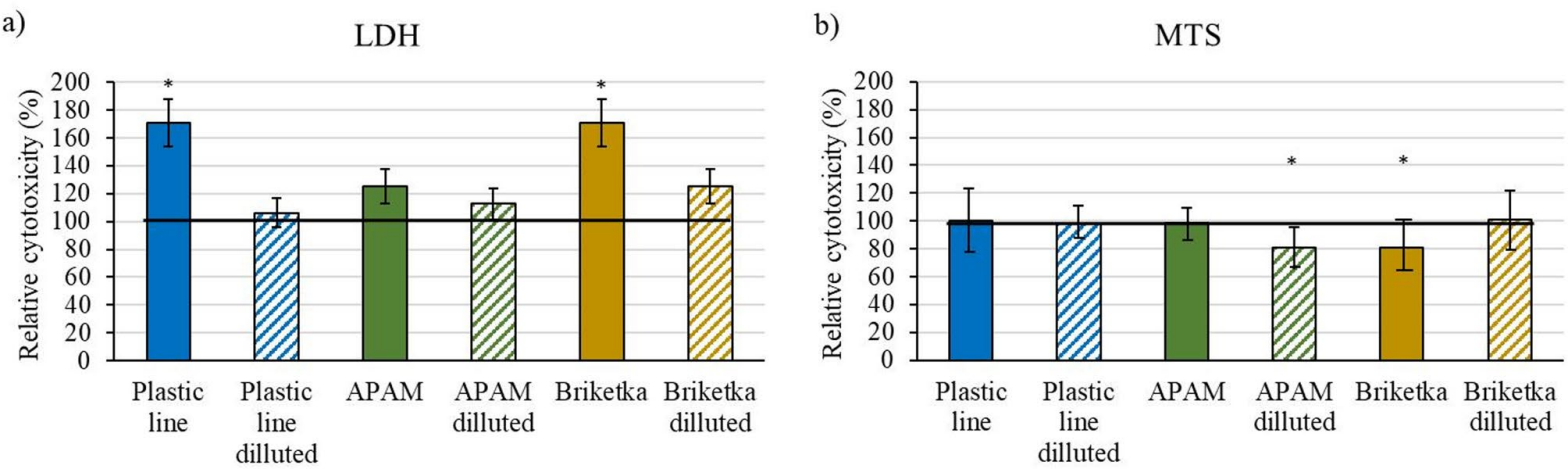
Determination of (**a**) LDH and (**b**) MTS in the undiluted and diluted extracts from filters sampled at the Plastic line, APAM and Briketka workplaces.

The undiluted extracts obtained from filters sampled in the Plastic line and Briketka workplaces, as well as the diluted extract obtained from a filter sampled in the APAM workplace, significantly affected cytotoxicity (Fig. 3). Compared with the control value (100), the extract obtained from the Plastic line increased the LDH release from the cells, indicating relative cytotoxicity (mean ± SD): 164.4 ± 23.6, *p* < 0.001. This could be due to a greater quantity of particles containing Ni, Cd and Co (see Table 1) trapped on the filter.

The effects of the extract obtained from the filter sampled in the APAM workplace were limited to the diluted sample. The MTS relative cytotoxicity activity compared to the control value (100) was found to be 85.5 ± 3.04 (mean ± SD), *p* < 0.05. Although the result is difficult to interpret, it suggests that the compounds extracted from this filter are relatively weakly toxic. This may be due to the higher amount of Fe present in the filters sampled in the Plastic line and APAM workplaces, which could have eliminated the negative impact of Cd^19^. Furthermore, higher concentrations of particles tend to agglomerate, which can shift the equilibrium^10^ and reduce the amount of Ni or Cd released from their compounds. The combined effect of these factors may have reduced the extract’s cytotoxic effect of the extract obtained from Plastic Line and APAM.

Compared with the control value of 100 (mean ± SD), the extract from Briketka significantly influenced the relative cytotoxicity of both LDH and MTS with values of 165.0 ± 20.1, *p* < 0.001 and 84.4 ± 6.39, *p* < 0.05, respectively. Increased LDH level and decreased MTS level suggests pronounced toxicity of the compounds adsorbed to the filter. Although the number of particles and Cd, Co and Ni contents were lowest for this sample, this sample may still have negative biological impacts due to the high proportion of Ni and Cd in the respirable fraction (see Fig. 1). The higher cytotoxicity of the filter sampled in the Briketka workplace could have resulted from a combination of several factors. The low concentrations of Ni and Cd (see Table 1) in the extract may have contributed to the better solubility of Ni(OH)_2_^10^ and Cd(OH)_2_, which probably contributed to the cytotoxicity by releasing Ni^2+^ and Cd^2+^ ions^26^. Therefore, it is not possible to determine exactly what caused the high cytotoxicity of the Briketka extract. Future studies investigating the combined effects of particle numbers, size, composition and simultaneously effect are desirable.

Overall, the compounds adsorbed onto the filters sampled in the Briketka workplace were the most toxic of all the tested samples and significantly impacted both LDH and MTS activities. The main cause of this toxicity may be the direct release of elemental Ni, Cd and adhesives from impregnated tapes during the production.

Although the metal concentrations determined on the filters were well below the maximum permissible concentrations and the concentration of Ni in the workplace environment is lower than the newly tightened limit, the extract from filters sampled in the workplace shows a negative impact on biological activity.

## Conclusion

The risk of exposure to potentially hazardous particles during Ni-Cd battery production was monitored at three selected workplaces considered to be the most at risk. Analysis showed that as the number of two-stage filters increased in the monitored workplaces, the number of particles trapped on the filters decreased. The respirable fraction (< 2 μm) was the most represented in all filters sampled at all workplaces. Although the concentrations of the monitored elements were well below the permissible exposure limits, the extracts from the filters collected at Plastic line and Briketka workplaces showed toxicity, which may be related to the high levels of Ni and Cd in the respirable fraction. Despite the high level of dust removal and very low concentrations of potentially hazardous elements on the filter, simultaneous toxicity tests indicate insufficient protection of workers’ health, especially in the case of long-term exposure. Based on the results, it can be concluded, that reducing of the permissible exposure limit may not provide sufficient protection against exposure to potentially hazardous particles in dust. In future, the toxicological assessments should be an essential part of the workplace monitoring, especially in the Plastic line and Briketka workplaces. Therefore, more sophisticated monitoring methods that focus on protecting workers’ health and incorporate more detailed toxicological data will be necessary to against the harmful effects of these toxicants.

## Materials and methods

###  Composition of Ni-Cd battery at the level of articles and modules

The active substances listed in Table 2 were mechanically enclosed between steel strips during battery production and were subsequently used to create electrodes. These electrodes were then assembled with the other components (separators, electrolyte, collection system, container) into finished batteries. According to the REACH regulation, batteries are defined as ‘products that do not release hazardous substances’, meaning that end users will not be exposed to the effects of chemical substances under normal and intended conditions of use.

**Table 2:** Composition of Ni-Cd battery at the level of Articles and modules. * Quantity varies by Article model.

Component		Content (wt%)*
Active nickel	Ni(OH)_2_ and NiOOH	4–15
Active cadmium	Cd(OH)_2_ and Cd	7–12
Cobalt	–	0–2
Alkaline electrolyte (pH = 14)	–	14–40
Plastics	–	5–20
Steel	–	10–40
Nickel	–	5–20
Copper	–	0–10

### Monitored workplaces

**Fig. 4:**
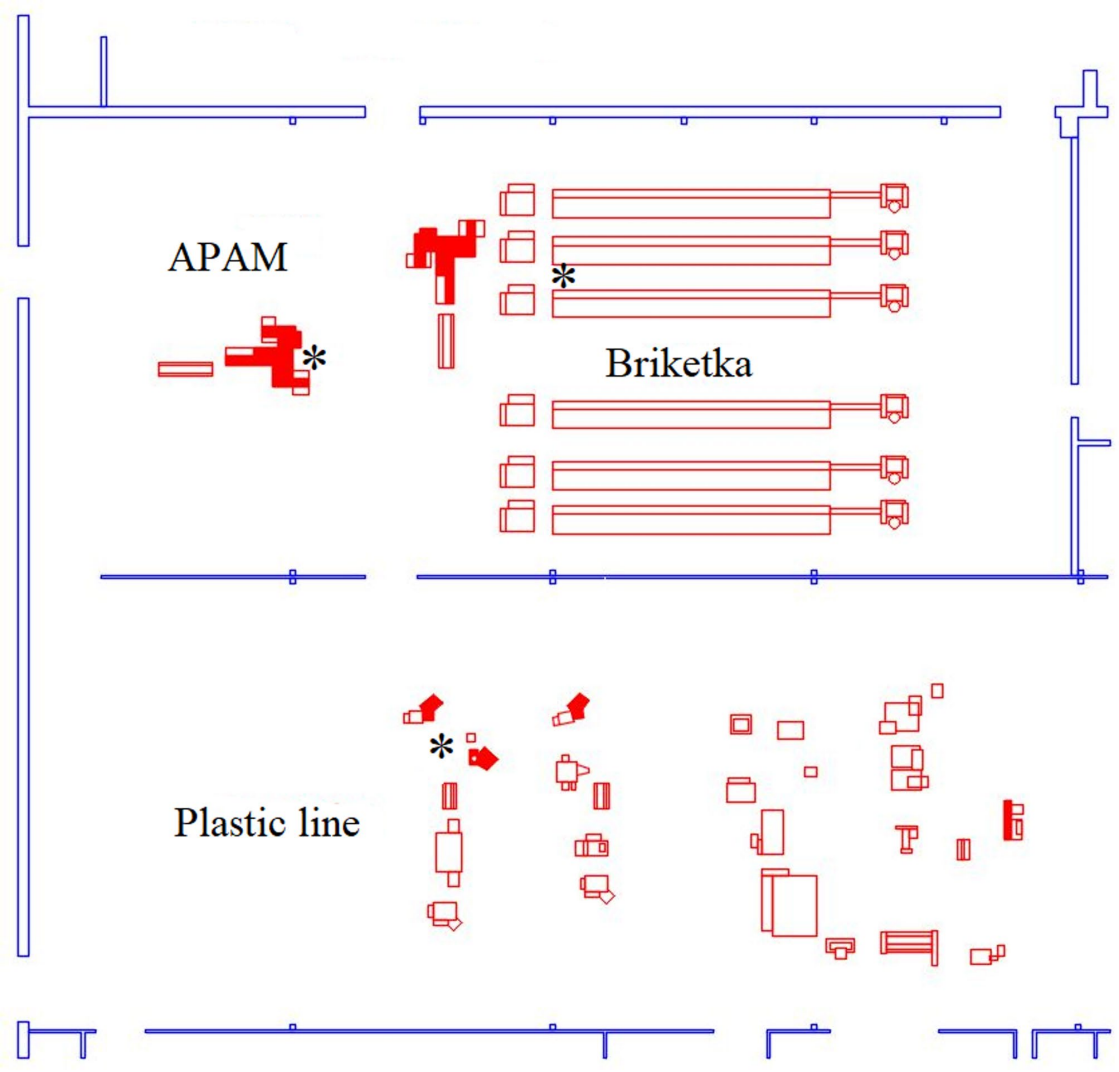
Diagram of the production hall with monitored workplaces: Plastic line, APAM and Briketka; * sampling sites.

Workplaces with the highest risk of exposure to dust containing heavy metals (nickel and cadmium) Briketka, APAM, Plastic line were selected for monitoring. The CARM GH (CIPRES FILTR BRNO s.r.o.) filter units were used to reduce the amount of aerosol pollutants, containing mainly Ni and Cd, in the production process. All filter types are designed as an automatically regenerating stationary filter units, intended for the most difficult filtration cases. The filter units are two-stage, with the first stage consisting of filter bags (pocket filters) which are automatically regenerated by compressed air (0.5–0.7 MPa) and are electronically controlled. In the upper part of the filter housing there are fans with a structural connection to the second filtration stage, called absolute filters AF (folded) or PAF (cartridges) and their regeneration is not possible. PAF cartridges can be cleaned by shaking and vacuuming with a vacuum cleaner or compressed air. The first stage of filtration ensures a purity of the filtered air of up to 5 mg/m^3^ for harmless solid substances. The second filtration stage ensures a purity of the filtered air down to 0.2 mg/m^3^ (for absolute filters) and 0.01–0.1 mg/m^3^ (PAF filters) for solid pollutants (Cd and Ni). The layout of the production hall is shown in Fig. 4.

Pockets are produced at a workplace called Briketka. There are 6 lines installed, each consisting of a briquetting press, a closing press, a worktable and a rolling machine with a plate cutter, with a maximum of two lines in operation at the same time. One line always processes only positive (containing Ni) or negative (containing Cd) pockets. Approximately 5300 pockets are produced in one work shift. These pockets are made from tapes that are impregnated with a substance containing nickel or cadmium and glue. The risk of contact with and possible inhalation of potentially hazardous particles is associated with handling the strips, which includes checking the weight, arranging the strips on the briquetting table, removing the produced pockets from the covered machine, tapping them and placing them in the shipping box. Nine two-stage filters are installed in this workplace.

On an APAM workstation, up to 3500 positive (nickel) or negative (cadmium) plates are machine-produced per shift. To prevent dust being blown into the air, the machine is completely enclosed by a plastic cover. There are two high-risk activities – plate handling and machine repair. The operator manually removes the produced plates from the machine, taps the plates against the suction pad to align them and places them in the transport box. The greatest risk of exposure to potentially hazardous dust occurs when the machine breaks down and the operator must open the machine, reach into a small confined space, bend over the machine, and, in most cases, wipe the detection sensors with a dry cloth to prevent moisture from entering the machine. There are 5 two-stage filters in the APAM workplace. At the time of the actual sampling, only one machine was in operation, which was operated by one employee.

A plastic line is used to produce the set. There are up to two operators. They rotate between the three stations every 20 min during their shift. The set is made from boards produced at the APAM workplace. Operators work with both negatively charged cadmium plates and positively charged nickel plates. The operator then assembles the boards, separating the formed nickel-cadmium sets with insulating separators before strapping them onto a machine. The handling of the boards is the biggest source of dust, with operators often tapping the table to align the boards or bending over them during welding and strapping. A two-stage filter is installed in the plastic line.

### Sampling

Sampling was carried out in accordance with the European standard EN 482:1994^27^. Aerosol sampling was performed using AirCheck pumps with an airflow rate of 2 L/min, tubing and IOM sampling heads fitted with 25 mm polycarbonate membrane filters with a porosity of 0.8 μm and an active filter area of 20 mm. Prior to sampling, a leak test was performed. Weighted polycarbonate filters were inserted into the IOM sampling heads, sealed with a lid and placed in a plastic container to prevent possible contamination during transport to the measurement site. The filters were placed in the operators´ breathing zone for the entire shift (6 a.m. to 1.45 p.m.), including safety and lunch breaks. The exposure time to dust containing potential carcinogens was 435 min. After sampling, the filters in the IOM sampling heads were transported to the laboratory in the plastic container and weighed prior to analysis.

During a standard shift, operators at the assigned workplaces collected a dust sample on one filter. Sampling was performed three times, with one filter collected from each workplace. The first series was obtained from sampling in the Plastic line, APAM and Briketka workplaces. This series was subjected to chemical analysis. The series carried out by second sampling was subjected to SEM, and a third series was subjected to cytotoxicity tests.

Although the filters were weighed immediately after sampling, the weight gain was less than the balance differentiation.

### Analysis of filter

Filters containing dust from several sampling points were dissolved in acid mixtures (aqua regia, nitric acid and hydrogen peroxide, analytical grade from MACH CHEMIKÁLIE s.r.o., Ostrava, Czech Republic) and the quantitative analysis was performed.

The concentrations of Cd, Co, Ni, Fe, Cr, Zn, V, Mo, Al, Ba, Be, Mn and Pb were determined using atomic emission spectrometry with inductively coupled plasma (OES-ICP) in End-On Plasma (EOP) observation mode Spectro Arcos (SPECTRO Analytical Instruments Inc., Germany).

The concentration of metals (in mg/m^3^) captured on the filter during sampling was calculated:

1 $$\:concentration\:of\:metal=\frac{{x}_{metal}}{v}$$

x_metal_ – amount of metal collected on the filter (mg/filter).

v – sampling rate (m^3^/hour).

Particle size distribution was analysed using a JEOL JSM-7610 F Plus scanning transmission electron microscope (STEM) with an Aztec Line standard microanalyser with an Ultim Max 65 analytical silicon drift detector (SDD). AZtec feature analysis software.

### Cytotoxicity test

Human bronchial epithelial cells (BEAS-2B) (ATCC, Manassas, VA, USA) were used for the cytotoxicity studies. Cells were grown in the BEBM medium (Lonza, Basel, Switzerland) supplemented with growth factors (BEGM™ BulleKit™, Lonza, Basel, Switzerland). BEAS-2B culture flasks were coated with 0.01 mg/ml human fibronectin (Sigma-Aldrich, St. Louse, Missouiri, United States), 0.03 mg/ml bovine collagen type I (Sigma-Aldrich, St. Louse, Missouiri, United States), and 0.01 mg/ml bovine serum albumin (BSA) in BEBM basal medium (Lonza, Basel, Switzerland) overnight in 37 °C.

Prior to testing, the filters were sonicated 3 times for 10 min in 5 ml of miliQ water. The total volume of 15 ml was divided into two parts into 15 ml test tubes. The resulting extracts were lyophilised, each of them dissolved in 1 ml of the BEGM medium, the content vigorously vortexed until completely dissolved. The solutions were combined and directly used for the cell treatment. For the samples denoted as “diluted” the extract was further diluted in the BEGM medium in the 1:1 ratio. For the control samples, clean, untreated filters were used; other steps were identical as described above. The cells plated at a concentration of 18,000 cells/well in 96-well plate were cultivated in medium containing the filter extracts (both undiluted and diluted 1:1 in BEGM medium) for 24 h. The cytotoxicity was measured using the MTS (Promega, Madison, WI, USA) and lactate dehydrogenase (LDH) (Roche, Basel, Switzerland) assays according to the manufacturer´s instructions. The samples were analysed in biological tetraplicates, each in technical duplicate. Absorbance was measured using a SpectraMax^®^ M5e plate spectrophotometer (Molecular Devices, San Jose, CA, USA) at a wavelength of 490 nm. The treated samples were compared with the controls using the two-tailed Student’s t-test, *p* ≤ 0.05 was considered significant.

## Data Availability

All data generated or analysed during this study are included in this published article.
